# Supplementary material for: Complex‐centric proteome profiling by SEC‐SWATH‐MS
Source: Mol Syst Biol. 2019 Jan 14;15(1):e8438. doi: 10.15252/msb.20188438 (PMC6346213; doi:10.15252/msb.20188438)
Supplement: Supplementary file 8 — Dataset EV7 [file MSB-15-e8438-s008.zip › feature_plots_string/P53618.pdf]

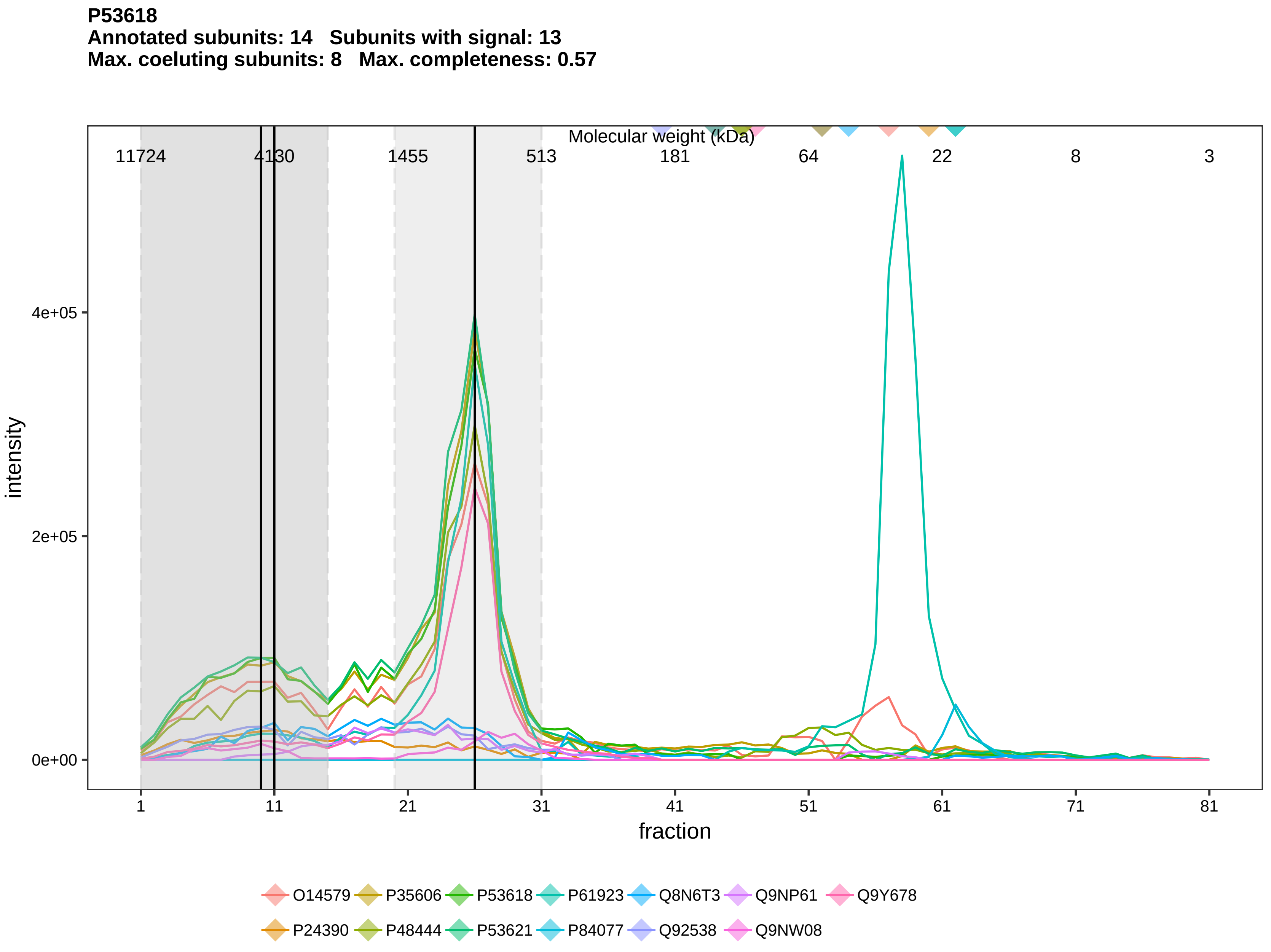
P53618
Annotated subunits: 14 Subunits with signal: 13
Max. coeluting subunits: 8 Max. completeness: 0.57
Molecular weight (kDa)
11724
4130
1455
513
181
64
22
8
3
intensity
4e+05
2e+05
0e+00
fraction
1
11
21
31
41
51
61
71
81
O14579
P35606
P53618
P61923
Q8N6T3
Q9NP61
Q9Y678
P24390
P48444
P53621
P84077
Q92538
Q9NW08
